# COVID-19-Vaccination-Induced Localized Lichen Planus Successfully Treated With Phototherapy

**DOI:** 10.7759/cureus.66907

**Published:** 2024-08-14

**Authors:** Mahdi Al Dhafiri

**Affiliations:** 1 Department of Dermatology, King Faisal University, Al-Ahsa, SAU

**Keywords:** covid-19, cutaneous manifestations of covid-19, covid-19 vaccine, post-phototherapy, lichen planus

## Abstract

Lichen planus (LP) is an inflammatory autoimmune mucocutaneous disease with different forms and presentations. It mainly affects the skin and oral mucosa but could also affect genital mucosa, nails, hair, and, rarely, the larynx and esophagus.

Since the start of the COVID-19 era, multiple cutaneous manifestations related to SARS-CoV-2 infection or vaccination have been reported. Different rare cases of lichen planus were reported after COVID-19 infection and vaccination.

This report elaborates on and adds an additional case of localized cutaneous lichen planus (CLP) to the upper extremities, which developed after both doses of the mRNA vaccine and improved after phototherapy.

## Introduction

Lichen planus (LP) is an uncommon inflammatory autoimmune papulosquamous pruritic polymorphic skin condition, affecting mainly middle-aged adults. The etiology is unknown, but different factors might play a role in developing this affection, including genetics, environmental factors, infection, and immune dysregulation [1,2].

The disease can present as violaceous pruritic flat-topped lesions symmetrical on the flexors of the extremities. LP can affect the skin, hair, nails, and mucous membranes, and oral presentation could be the only manifestation of the disease [3].

Multiple mucocutaneous reactions have been reported since the start of the COVID-19 era. Moreover, multiple reactions have been reported after administering the different mRNA vaccines. Different cases of LP were reported after COVID-19 infection and vaccination [4,5].

Herein, we discuss a case of localized LP that started after the mRNA vaccine on the dorsum of both hands and improved after multiple phototherapy sessions.

## Case presentation

**Figure 1:**
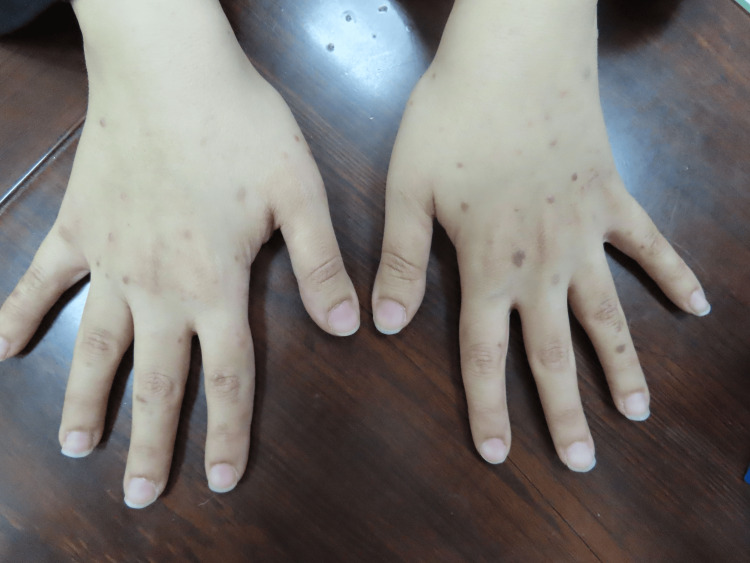
Eruptive lesions of lichen planus on the upper extremities

**Figure 2:**
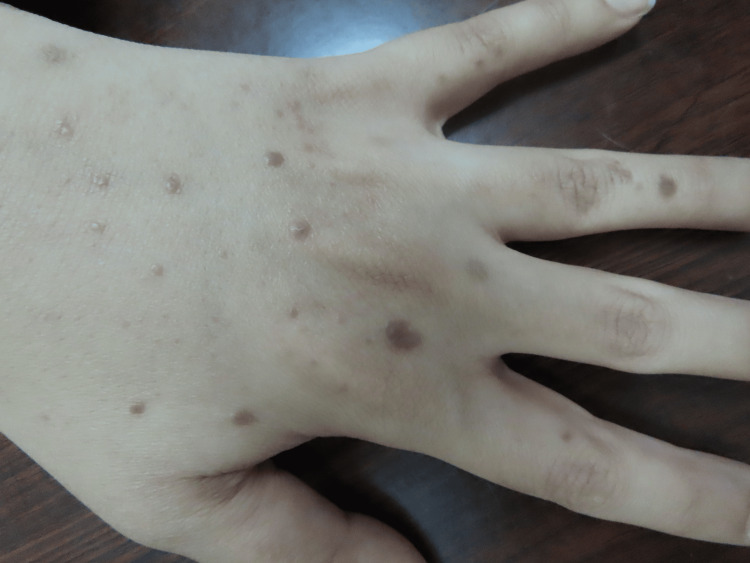
Multiple lesions of lichen planus on the dorsum of the left hand

**Figure 3:**
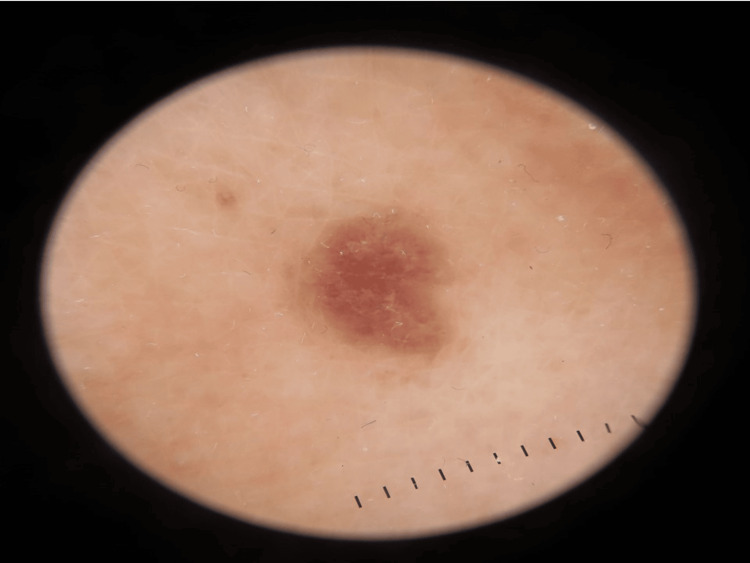
A dermoscopic image showing the characteristic Wickham striae

A 37-year-old female patient presented to our clinic two weeks after receiving the mRNA vaccine BNT162b2 (Pfizer). She started to develop multiple violaceous hyperpigmented flat-topped with fine scaling pruritic papules on the dorsum of the hands bilaterally (Figures 1, 2). After three months, the patient received a second booster immunization, and then after one week, she developed more additional skin lesions on the same localization symmetrical on both hands. Based on the clinical image, symptoms, and dermoscopic picture showing the characteristic Wickham striae (Figure 3), cutaneous lichen planus (CLP) was diagnosed.

Different topical corticosteroids were used with no/partial improvement, including mometasone furoate cream, clobetasol propionate cream, and betamethasone dipropionate cream. After that, treatment with topical tacrolimus 0.1% ointment was started once daily, in addition to weekly sessions of targeted broadband ultraviolet B (UVB) phototherapy; excellent improvement and complete remission of the lesions after two months of this treatment were achieved. No recurrence was reported.

## Discussion

Lichen planus (LP) affects almost <1% of the general population, of both genders and all ethnicities, and the age of onset is generally between 30 and 60 years with rare pediatric cases. It is represented as flat-topped papules-plaques that are violaceous and shiny, with fine scaling and the presence of the characteristic Wickham striae. It affects mainly the extremities, but generalized presentation is possible. LP can resolve within one to two years, but mucous presentation can have a more chronic course [1-3].

Lichen planus is generally diagnosed clinically. However, a histopathologic image represents a confirmative measure, and it shows hyperkeratosis with apoptotic keratinocytes and hypergranulosis with a "saw tooth" appearance in the rete ridges in the epidermis and a small cleft in the dermo-epidermal junction with lymphocyte infiltration and pigment incontinence. Direct immunofluorescence examination shows a linear "shaggy" band of fibrinogen deposition at the basement membrane, as well as colloid bodies in the papillary dermis staining with any of the autoantibody immunoglobulins [1,2]. Using dermoscopy can help confirm the presence of the characteristic Wickham striae [1,3].

Cutaneous LP (CLP) can have different morphological presentations, including atrophic, pigmented, hyperkeratosis, linear, inverse, actinic, follicular, or bullous. Lichen planus can also affect other areas, including nails, mucous membranes (oral and genital mainly), and the scalp [1-3].

The pathogenesis of LP is still not well understood. However, certain factors are believed to play an important role in the etiology, and that includes immune dysregulation; infections, mainly hepatitis C virus; environmental factors such as medications and stressful events; and genetic predisposition [1,3,6].

Several therapeutic options for LP are available, including phototherapy, topical corticosteroids, and calcineurin inhibitors. A systemic therapy could be considered for recurrent, refractory, and diffused CLP, and it includes mainly systemic corticosteroids, retinoids such as acitretin, low-molecular-weight heparin, immunosuppressant drugs such as methotrexate, azathioprine, and cyclosporin, immunomodulatory drugs such as sulfasalazine and thalidomide, hydroxychloroquine, and biologics [7].

Since the start of the COVID-19 pandemic in December 2029, several cutaneous manifestations have been linked to the infection with coronavirus, including vasculitic eruption, mainly chilblain-like skin eruption, purpura, livedo reticularis, and necrotic lesions, and inflammatory skin eruptions, including urticaria-like eruptions, vesicular eruptions, and maculopapular lesions. Other manifestations have been additionally reported, including the increased incidence of herpes zoster and alopecia [8,9].

The exact pathogenesis for the COVID-19-induced skin manifestations still needs to be better understood. However, for the vasculitic eruption, there was the perivascular identification of SARS-CoV-2 spike protein, angiotensin-converting enzyme 2 (ACE2)-mediated endothelial damage, and increased levels of inflammatory cytokines such as IL-6. Moreover, it is considered that the inflammatory skin manifestation is caused by the high level of cytokines induced by COVID-19, resulting in the perivascular infiltration of inflammatory cells, vasodilatation, and edema [8].

Nevertheless, different skin affections have been linked to mRNA-based COVID-19 vaccination. The cutaneous reactions were reported with the different mRNA vaccines, including BNT162b2 (Pfizer/BioNTech), mRNA-1273 (Moderna), and AZD1222 (AstraZeneca). The reactions include type I hypersensitivity reactions such as urticaria, angioedema, and uncommon cases of anaphylaxis. The exact mechanism is not known. However, the vaccine contains certain substances known to develop allergic reactions, such as polyethylene glycol (PEG), polysorbate 80, and tromethamine/trometamol. Type IV hypersensitivity is another reaction, which includes mainly a delayed localized skin reaction on the site of vaccination, "COVID arm." It starts about seven days after the first immunization and about two days after the second immunization and disappears after about three days. It is represented as edema, erythema, and induration. Moreover, this reaction has also been linked to edema at the previous Bacillus Calmette-Guérin (BCG) vaccination site and facial edema for those who underwent filler injections for cosmetic purposes. It is also linked to causing a morbilliform erythema multiforme-like reaction on the face, trunk, and extremities. Also, there is an autoimmune-mediated reaction with reported cases of flare of lupus erythematosus, vasculitis, purpura, vitiligo, and alopecia areata, and the most probable reason is that the spike protein of the COVID-19 vaccine is similar to the human protein, which can induce the autoimmune reaction. Additionally, different cases of maculopapular eruption, the reactivation of herpes viruses 6 and 7 causing pityriasis rosea, and the reactivation of herpes zoster were reported in different countries [4,8-10].

Different cases of LP have been reported as a rare adverse reaction/complication after COVID-19 vaccination. Different hypotheses discuss the possible causative mechanism, including that the spike protein of the COVID-19 vaccine stimulates the T cells, producing proinflammatory cytokines that trigger the basal keratinocytes of the epidermis and the formation of LP. Others suggest that vaccines stimulate cluster of differentiation 4+ (CD4+) type 1 helper T cells (Th1), leading to the production of inflammatory mediators, including cytokines, interferon-α, and tumor necrosis factor-α, leading to basal keratinocytes apoptosis and the formation of LP. Moreover, others suggest that COVID-19 spike protein stimulates ACE2 receptors on the epidermal cells that trigger Th1, leading to an autoimmune reaction causing the formation of LP lesions. Others suggest that the reticuloendothelial system might induce a COVID-19-vaccine-induced hyperinflammatory reaction leading to the formation of LP [5,11].

In this report, we present a case of a young female patient with multiple lesions of LP localized on the dorsum symmetrically on both hands. The patient got a new flare after the second immunization. The lesions improved and healed after targeted UVB phototherapy.

## Conclusions

Since the start of the COVID-19 pandemic, multiple skin reactions and lesions have been described and reported. Since the administration of the mRNA vaccine in December 2020, additional multiple skin reactions have been reported as adverse effects of the immunization, including cases of LP. COVID-19-induced LP is still a rare adverse reaction, and it should not prevent patients from getting the vaccination, as the benefits outweigh the risks. Phototherapy is a treatment to be considered especially in the case of ineffective topical options.
